# The sports-education-health nexus: assessing psychological, cognitive, and social outcomes in a public health framework

**DOI:** 10.3389/fpubh.2026.1859935

**Published:** 2026-06-15

**Authors:** Bo Ling

**Affiliations:** College of Physical Education, Changzhou University, Changzhou, China

**Keywords:** cognitive focus, emotional intelligence, psychological well-being, social development, sports education, strategic capacity

## Abstract

This study investigates the multidimensional relationships between sports education and psychological, cognitive, social, strategic, and environmental outcomes. The purpose of the study is to examine the multidimensional role of sports education in shaping psychological, cognitive, social, strategic, and environmental outcomes, while also assessing the mediating roles of psychological well-being and strategic capacity. A quantitative research design was adopted, and data were analyzed using Partial Least Squares Structural Equation Modeling (PLS-SEM). The findings show that sports education has a significant positive effect on social development, psychological well-being, environmental awareness, cognitive focus, and strategic capacity. The results further indicate that psychological well-being positively influences cognitive focus, whereas strategic capacity positively affects social development. In addition, psychological well-being mediates the relationship between sports education and cognitive focus, while strategic capacity mediates the relationship between sports education and social development. Emotional intelligence was included as an additional construct and was found to have significant positive effects on psychological well-being and cognitive focus, although its direct effect on social development was not statistically significant. Overall, the study highlights the multidimensional developmental value of sports education, particularly in promoting psychological well-being, cognitive focus, social development, strategic capacity, and environmental awareness across diverse learning contexts.

## Introduction

Sports education refers to the structured process of learning and teaching physical activities, sports, and physical education within academic and institutional settings (1). It encompasses not only the development of physical skills but also contributes to cognitive, emotional, and social competencies through active participation in sports-related activities (2). As an interdisciplinary domain, sports education plays a crucial role in fostering human development, promoting social cohesion, and enhancing overall well-being. It encourages active lifestyles, reduces health risks, and equips individuals with essential life skills such as teamwork, discipline, communication, and time management (3).

Sports education has been widely recognized as a key contributor to multiple dimensions of development, including psychological, social, cognitive, and environmental outcomes (4). In particular, psychological well-being (PW) represents an essential aspect of human functioning, encompassing emotional stability, self-esteem, and stress resilience. Participation in sports activities has been shown to improve psychological well-being by reducing anxiety, enhancing emotional regulation, and fostering a sense of achievement (5). These benefits are particularly important in contemporary educational environments, where individuals face increasing academic and social pressures (6). Meta-analysis studies conducted in Türkiye have reported that physical activity levels differ according to the gender variable and demonstrate a higher effect size in favor of males (7). This finding indicates that gender may be an important determinant in evaluating the psychological, social, and cognitive outcomes of sports education.

Rather than focusing exclusively on physical health outcomes, the present study adopts a broader developmental perspective by examining the psychological, cognitive, social, strategic, and environmental dimensions associated with sports education participation. In addition to psychological well-being, sports education significantly contributes to social development (SD), which refers to individuals’ ability to interact effectively within society. Through team-based activities and collaborative participation, sports education promotes communication, leadership, cooperation, and interpersonal skills (8). These social competencies are critical for building strong relationships and fostering social cohesion within communities (9). Similarly, cognitive focus (CF), defined as the ability to sustain attention and process information efficiently, is enhanced through sports participation (10). Engaging in sports requires individuals to make quick decisions, adapt to dynamic situations, and maintain concentration, thereby improving cognitive performance and mental agility (11).

Psychological well-being and cognitive focus in sports environments are associated not only with sports education but also with the individual’s motivational structure and family support. Research conducted on amateur athletes in Türkiye has indicated that achievement motivation and parental attitudes create significant effects on sports performance (12).

Furthermore, sports education plays an important role in developing strategic capacity (SC), which involves the ability to plan, organize, and execute effective strategies to achieve specific goals (13). Participation in sports activities fosters strategic thinking, problem-solving, and decision-making skills, which are transferable to academic and professional contexts. Another important outcome of sports education is environmental awareness (EA), which reflects individuals’ understanding and concern for environmental issues (14). Sports activities, particularly those conducted in outdoor settings, provide opportunities for individuals to interact with natural environments, thereby promoting environmental consciousness and sustainable behavior (15).

Despite the growing body of literature highlighting the benefits of sports education, existing research has primarily examined these outcomes in isolation, without fully exploring the integrated relationships among psychological well-being, social development, cognitive focus, strategic capacity, and environmental awareness (16). Moreover, the mechanisms through which sports education influences these outcomes remain insufficiently understood (17). In particular, the mediating roles of psychological well-being in the relationship between sports education and cognitive focus, and strategic capacity in the relationship between sports education and social development, require further investigation (4).

In addition, recent developments in behavioral sciences suggest that emotional intelligence (EI) plays a significant role in enhancing individual performance across psychological, cognitive, and social domains (18). Emotional intelligence refers to the ability to perceive, understand, and regulate emotions effectively. It is likely to strengthen the impact of sports education by improving emotional regulation, interpersonal relationships, and cognitive functioning (19). However, the role of emotional intelligence within the sports education framework has not been sufficiently explored, representing an important avenue for future research (20).

Based on these gaps, the present study aims to develop an extended conceptual framework that examines the relationships among sports education, psychological well-being, social development, cognitive focus, strategic capacity, and environmental awareness, while incorporating emotional intelligence as an additional construct. Specifically, this study seeks to:

- Examine the impact of sports education (SE) on psychological well-being (PW), social development (SD), cognitive focus (CF), strategic capacity (SC), and environmental awareness (EA).
- Investigate the mediating role of psychological well-being (PW) in the relationship between sports education (SE) and cognitive focus (CF).
- Analyze the mediating role of strategic capacity (SC) in the relationship between sports education (SE) and social development (SD).
- Explore the role of emotional intelligence (EI) in enhancing psychological well-being and cognitive outcomes.

This study contributes to the literature by providing a comprehensive framework that integrates multiple dimensions of sports education outcomes. It extends existing research by incorporating emotional intelligence as a key variable and by examining the interconnected relationships among psychological, cognitive, social, and environmental factors. The findings are expected to provide valuable insights for educators, policymakers, and practitioners, emphasizing the importance of sports education in promoting holistic development and sustainable societal outcomes.

### Conceptual framework and hypotheses

Sports education plays a fundamental role in shaping multiple dimensions of individual and societal development, particularly social development, psychological well-being, cognitive focus, strategic capacity, and environmental awareness (21). It provides a structured and interactive environment in which individuals engage in physical, cognitive, and social activities, facilitating learning through participation, observation, and experience (22). The theoretical foundation of this relationship is supported by Social Learning Theory, which posits that individuals acquire behaviors, skills, and attitudes through interaction and observation within structured environments (23). Sports education offers such a platform where individuals develop teamwork, communication skills, emotional resilience, and cognitive abilities that contribute to overall development (1).

Sports education is essential in fostering social development (SD), as it enhances individuals’ ability to collaborate, communicate effectively, and function within diverse social environments (24). Participation in team-based sports encourages cooperation, leadership, and interpersonal interaction, which are critical for building social relationships and community cohesion (25). Social Capital Theory further emphasizes that trust, shared values, and social networks are key drivers of collective well-being, and sports education contributes to building these elements by promoting interaction and shared experiences (26). Empirical evidence suggests that individuals involved in sports activities exhibit stronger social skills, better communication abilities, and higher levels of social integration compared to non-participants (27).

*H1:* Sports education has a positive impact on social development by fostering teamwork, enhancing communication skills, and promoting social interaction and integration among individuals.

Sports education also plays a significant role in enhancing psychological well-being (PW), which encompasses emotional stability, self-esteem, and stress management. According to Self-Determination Theory, individuals achieve higher levels of well-being when their psychological needs for autonomy, competence, and relatedness are fulfilled (28). Sports education satisfies these needs by providing opportunities for skill development, social bonding, and active participation in meaningful activities. Participation in sports has been associated with reduced stress, improved mood, and increased emotional resilience, which are essential for maintaining mental health (4).

*H2:* Sports education has a positive impact on psychological well-being by reducing stress, improving self-esteem, and promoting emotional stability and resilience.

Environmental awareness (EA) is another important outcome influenced by sports education. Participation in outdoor sports and physical activities exposes individuals to natural environments, fostering ecological sensitivity and environmental responsibility (29). Experiential Learning Theory suggests that learning through direct experience enhances individuals’ understanding of environmental issues and promotes sustainable behavior. Sports education programs that incorporate environmental themes further strengthen individuals’ awareness of sustainability and encourage environmentally responsible actions (30).

*H3:* Sports education has a positive impact on environmental awareness by encouraging interaction with natural environments, promoting sustainable practices, and enhancing ecological responsibility.

Cognitive focus (CF), defined as the ability to sustain attention and process information effectively, is significantly enhanced through sports participation (10). The Embodied Cognition Theory highlights the connection between physical activity and cognitive processes, suggesting that movement-based activities improve mental functioning. Sports require individuals to make quick decisions, anticipate outcomes, and adapt to dynamic situations, which strengthens attentional control, working memory, and problem-solving abilities. Empirical studies have demonstrated that individuals engaged in sports exhibit improved concentration and cognitive efficiency (31).

*H4:* Sports education has a positive impact on cognitive focus by improving attentional control, enhancing information processing, and strengthening mental clarity and concentration.

Strategic capacity (SC), which refers to the ability to plan, organize, and make effective decisions, is also developed through sports education (32). Participation in sports requires individuals to analyze complex situations, formulate strategies, and adapt to changing environments (33). Cognitive Load Theory explains that engaging in complex tasks enhances individuals’ ability to process information efficiently and make informed decisions. These strategic skills are transferable to academic and professional settings, contributing to overall development (34).

*H5:* Sports education has a positive impact on strategic capacity by developing decision-making skills, promoting planning abilities, and enhancing adaptability in dynamic situations.

Psychological well-being plays a critical role in influencing cognitive focus. The Broaden-and-Build Theory suggests that positive emotional states expand individuals’ cognitive capabilities, enabling better attention, flexibility, and problem-solving abilities (35). Conversely, stress and emotional instability reduce cognitive efficiency by increasing cognitive load. Individuals with higher psychological well-being are more likely to maintain focus, process information effectively, and perform better in cognitively demanding tasks (36).

*H6:* Psychological well-being has a positive impact on cognitive focus, as emotional stability and reduced stress levels contribute to improved attention, mental clarity, and sustained task engagement.

Strategic capacity also contributes significantly to social development. Individuals with strong strategic skills are better equipped to plan, collaborate, and contribute effectively within social and organizational contexts (37). Human Capital Theory emphasizes that individuals with higher competencies are more capable of contributing to societal development (38). Strategic capacity enables individuals to organize collective actions, coordinate activities, and achieve shared goals, thereby enhancing social cohesion (39).

*H7:* Strategic capacity has a positive impact on social development by enhancing individuals’ ability to plan, collaborate, and effectively engage in social and community-oriented activities.

Psychological well-being serves as an important mediating mechanism between sports education and cognitive focus (4). Participation in sports improves emotional stability and reduces stress, which in turn enhances cognitive functioning. Individuals with better psychological well-being are more capable of allocating cognitive resources efficiently, resulting in improved attention and mental clarity (40).

*H8:* Psychological well-being mediates the relationship between sports education and cognitive focus, such that improvements in emotional stability and stress reduction enhance cognitive performance and attention.

Similarly, strategic capacity mediates the relationship between sports education and social development (41). Sports education enhances individuals’ strategic thinking and decision-making abilities, which subsequently improve their ability to interact socially and contribute to community development. This highlights the indirect pathways through which sports education influences social outcomes (42).

*H9:* Strategic capacity mediates the relationship between sports education and social development, whereby enhanced planning and decision-making abilities improve social interaction and community engagement.

In line with emerging perspectives in behavioral and social sciences, emotional intelligence (EI) has been recognized as a critical factor influencing psychological, cognitive, and social outcomes (43). Emotional intelligence refers to the ability of individuals to perceive, understand, regulate, and effectively manage their own emotions as well as the emotions of others. It plays a vital role in shaping how individuals respond to challenges, interact with others, and perform in both academic and real-life situations (44). The integration of emotional intelligence into the sports education framework provides a more comprehensive understanding of the psychological and behavioral mechanisms underlying individual development (45).

The theoretical foundation of emotional intelligence is supported by Emotional Intelligence Theory, which emphasizes that individuals with higher emotional intelligence possess better emotional regulation, self-awareness, and interpersonal skills (46). These abilities enable individuals to manage stress effectively, maintain emotional stability, and respond adaptively to complex situations (47). In addition, the Affective Events Theory suggests that emotional experiences significantly influence individuals’ attitudes, behaviors, and performance outcomes (18). Within the context of sports education, emotionally intelligent individuals are better equipped to handle competitive pressure, maintain motivation, and sustain positive emotional states, which contribute to improved psychological well-being (4).

Emotional intelligence is closely linked to psychological well-being (PW), as individuals with higher emotional intelligence are more capable of regulating negative emotions and enhancing positive emotional experiences (48). They are better able to cope with stress, reduce anxiety, and maintain emotional balance, which leads to improved mental health outcomes (49). Empirical evidence suggests that emotional intelligence significantly contributes to higher self-esteem, emotional resilience, and life satisfaction. In sports contexts, emotionally intelligent individuals are more likely to remain composed under pressure, manage performance-related stress, and maintain a positive mindset (50).

*H10:* Emotional intelligence has a positive impact on psychological well-being by improving emotional regulation, enhancing self-awareness, and enabling individuals to effectively manage stress and emotional challenges, thereby promoting emotional stability and overall mental health.

Emotional intelligence also plays a crucial role in enhancing cognitive focus (CF). Individuals with higher emotional intelligence are better able to regulate their emotional states, which reduces cognitive distractions and improves attentional control (50). The Cognitive Resource Theory suggests that emotional regulation allows individuals to allocate cognitive resources more efficiently, leading to improved concentration and information processing. In both academic and sports contexts, emotionally intelligent individuals are more capable of maintaining focus under pressure and sustaining mental clarity (51).

*H11:* Emotional intelligence has a positive impact on cognitive focus by facilitating better emotional control, reducing cognitive distractions, and improving individuals’ ability to concentrate, process information efficiently, and maintain sustained attention in challenging situations.

Furthermore, emotional intelligence significantly contributes to social development (SD), as it enhances individuals’ ability to understand and respond to the emotions of others (18). Social Interaction Theory suggests that effective communication and interpersonal relationships are largely influenced by emotional understanding and empathy (19). Individuals with higher emotional intelligence are better able to build relationships, resolve conflicts, and engage in cooperative behaviors. In sports settings, emotional intelligence promotes teamwork, mutual understanding, and effective communication, which are essential for successful group performance (19).

In contrast, H12 was not supported, indicating that emotional intelligence does not have a statistically significant direct effect on social development within the present model. This finding suggests that social development in sports education contexts may depend more strongly on direct interpersonal experiences, collaborative participation, teamwork dynamics, and strategic interaction than on emotional intelligence alone. Although emotionally intelligent individuals may demonstrate stronger emotional regulation and self-awareness, these capabilities may not automatically translate into broader social development unless they are reinforced through active social engagement and structured group interaction.

The finding also indicates that strategic capacity and sports education may play more dominant roles in shaping social development because sports environments inherently require cooperation, coordination, tactical adaptation, and collective participation. In this context, strategic capacity may function as a more immediate mechanism for improving social functioning, whereas emotional intelligence may exert a more indirect influence through psychological well-being, communication quality, or interpersonal adjustment. Therefore, the non-significant result does not diminish the importance of emotional intelligence; rather, it suggests that its contribution to social development may be context-dependent and mediated by other behavioral or social processes.

### Methodology

#### Measures

The sampling framework was designed to ensure representation across different demographic categories, including gender, educational level, and rural urban background. The inclusion of respondents from diverse demographic groups was considered important because participation in sports education and its developmental outcomes may vary across social and educational contexts. A heterogeneous sample therefore improves the representativeness and generalizability of the findings while also strengthening the robustness of the structural model assessment.

The questionnaire items used to measure the study constructs were adopted from previously validated scales reported in earlier studies in order to ensure content validity, construct reliability, and consistency with prior empirical research (Table 1).

**Table 1:** Sources of adapted measurement scales.

Construct	Number of items	Adapted from
Sports Education (SE)	5	Yang et al. (55)
Psychological Well-being (PW)	5	Park et al. (57)
Social Development (SD)	5	Liqiang et al. (25)
Cognitive Focus (CF)	5	Walters et al. (60)
Strategic Capacity (SC)	5	Müller et al. (62)
Environmental Awareness (EA)	5	Baltodano-Nontol et al. (58)
Emotional Intelligence (EI)	5	Yang and Duan (63)

Constructs such as sports education, psychological well-being, social development, cognitive focus, strategic capacity, environmental awareness, and emotional intelligence were operationalized using a five-point Likert scale ranging from 1 = strongly disagree to 5 = strongly agree. A five-point scale was adopted as it is widely recommended for improving response quality and reducing respondent fatigue. This study employed a cross-sectional research design and a quantitative approach to examine the relationships among the study variables.

**Figure 1:**
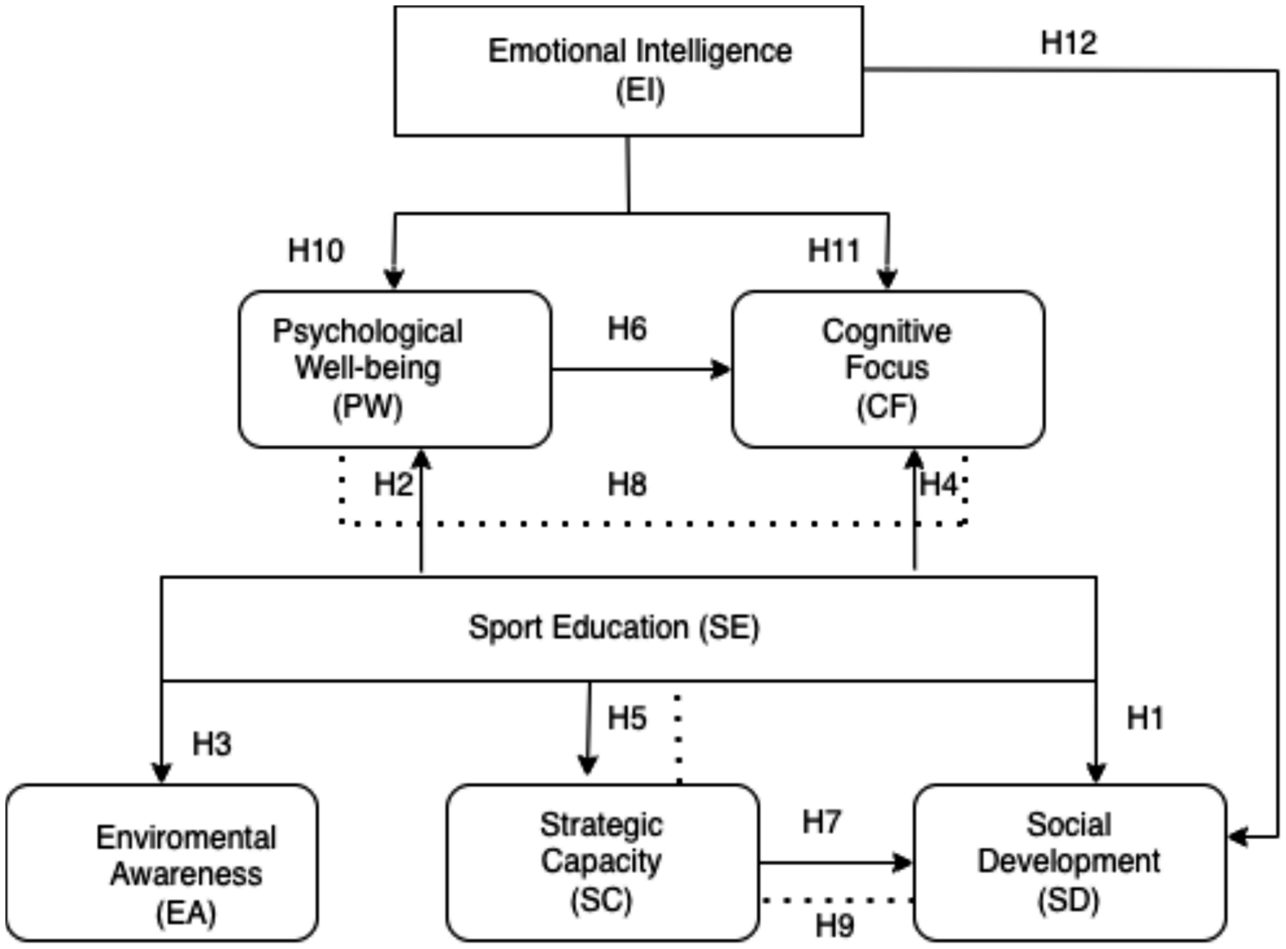
Conceptual framework of the proposed research model. Solid arrows represent direct hypothesized relationships, whereas dotted arrows indicate mediation pathways within the conceptual framework.

To ensure content validity and contextual relevance, the adapted measurement items were reviewed by academic experts with expertise in sports education and behavioral research. Following expert evaluation, a pilot test was conducted with a small group of respondents to assess clarity, wording, and comprehensibility of the questionnaire items (52). Based on the feedback obtained, minor modifications were made to improve readability and eliminate ambiguity (53). The final questionnaire demonstrated satisfactory reliability and was considered suitable for full-scale data collection. The proposed conceptual framework illustrating the hypothesized relationships among the study variables is presented in Figure 1.

Sports education is conceptualized as a multidimensional construct reflecting participation, engagement, and the perceived effectiveness of structured sports activities within an educational context (4). It encompasses the learning and teaching processes related to physical education, sports participation, and skill development, contributing to cognitive, emotional, and social growth (54). The measurement of sports education was based on five indicators capturing individuals’ involvement in sports activities, the quality of sports programs, and the extent to which sports education enhances personal development, teamwork, and physical well-being (55).

Social development was measured using items adapted from prior studies focusing on interpersonal and social competencies. It refers to the ability of individuals to effectively interact, collaborate, and function within a social environment (56). The construct includes dimensions such as teamwork, leadership, communication, and social interaction. The measurement items assess the extent to which participation in sports enhances individuals’ social skills, strengthens interpersonal relationships, and promotes a sense of belonging and community engagement (25).

Psychological well-being was operationalized using items that capture emotional stability, self-esteem, and overall mental health. This construct reflects individuals’ ability to manage stress, maintain positive emotions, and achieve a balanced psychological state (57). The measurement items were designed to allow respondents to evaluate their mental well-being in relation to their participation in sports activities, including the extent to which sports contribute to emotional resilience and life satisfaction (4).

Environmental awareness was measured using items that assess individuals’ understanding and concern for environmental issues, particularly in relation to sports participation. The construct captures awareness of environmental sustainability, appreciation of natural surroundings, and recognition of the importance of eco-friendly practices (58). Participation in sports, especially outdoor activities, is expected to enhance individuals’ environmental consciousness and promote responsible behavior toward the environment (15).

Cognitive focus was measured by capturing individuals’ ability to concentrate, sustain attention, and remain mentally engaged during activities. The construct reflects attentional control, mental clarity, and the ability to process information effectively (59). The measurement items assess how participation in sports activities influences individuals’ capacity to maintain focus, avoid distractions, and remain cognitively engaged during both physical and academic tasks (60).

Strategic capacity was measured using indicators that reflect individuals’ ability to plan, make decisions, and adapt to changing situations. It represents the cognitive and behavioral ability to develop and implement effective strategies in dynamic environments (61). The measurement items evaluate how participation in sports enhances individuals’ strategic thinking, problem-solving skills, and ability to respond to challenges in both sports and real-life contexts (62).

Emotional intelligence refers to individuals’ ability to perceive, understand, and regulate their own emotions as well as the emotions of others. It plays a critical role in shaping psychological, cognitive, and social outcomes (63). The measurement items capture key dimensions of emotional intelligence, including emotional regulation, self-awareness, empathy, and interpersonal understanding. The inclusion of emotional intelligence extends the existing framework and provides a more comprehensive understanding of the mechanisms through which sports education influences individual development (19).

#### Respondents

The study was conducted in accordance with accepted ethical standards for research involving human participants. Written informed consent was obtained from all respondents before participation in the survey. The questionnaire was distributed through email, academic networks, and social media platforms. Data were collected over a four-month period, from February 1, 2024, to May 30, 2024. The study was conducted in selected universities and colleges in Changzhou, Jiangsu Province, China. This setting was appropriate because sports education and organized physical activity programs are actively embedded in educational institutions, providing a relevant context for examining the effects of sports education on psychological well-being, cognitive focus, social development, strategic capacity, environmental awareness, and emotional intelligence among adult participants. The population of interest comprised individuals aged 18 years and above who had participated in sports education activities or were actively involved in sports-related programs. A convenience sampling technique with stratified outreach was employed to recruit respondents from different regions, ensuring variation across gender, education level, and residential background (64). In total, 950 questionnaires were distributed, of which 742 were found suitable for final analysis. Responses that were incomplete, inconsistent, or failed to meet the study requirements were excluded from further analysis (65). To ensure data quality, several screening procedures were applied. Duplicate submissions were identified through response patterns, timestamps, and repeated contact identifiers, while responses with substantial missing values, straight-lining behavior, or unusually short completion times were removed (66). In addition, only respondents who satisfied the eligibility criteria namely being adults, having prior exposure to sports education or related sports participation, and completing the questionnaire in full were retained in the final dataset (67). To reduce self-selection and non-response bias, the survey link was shared across diverse channels targeting participants from different demographic categories (68). Furthermore, anonymity and confidentiality were emphasized in order to encourage honest and complete responses. These procedures helped ensure that the final dataset was valid and appropriate for empirical analysis.

The final sample size of *N* = 742 was considered adequate for structural equation modeling analysis. In line with established sample adequacy recommendations, the final number of usable responses exceeded the minimum threshold required for reliable model estimation (69). The demographic profile of the respondents included 54% male and 46% female, 64% undergraduate and 36% postgraduate, and 58% urban and 42% rural participants, indicating that the sample represented a reasonably diverse cross-section of the target population. This diversity strengthens the validity and generalizability of the study findings. Since the study relied on self-reported survey responses, the possibility of common method bias (CMB) was also considered. Previous studies have shown that CMB may artificially inflate the observed associations among variables (70). Therefore, both procedural and statistical remedies were adopted. First, the questionnaire items were designed in a clear and concise manner to reduce ambiguity and misunderstanding. Second, respondents were assured that their information would remain confidential and would be used only for academic purposes, thereby minimizing social desirability bias (71). Third, Harman’s single-factor test was conducted to assess common method variance (72). All measurement items were entered into an exploratory factor analysis using unrotated principal component analysis (73). The results indicated that the first factor accounted for 31.2% of the total variance, which is below the recommended threshold of 50%. This suggests that common method bias was not a serious concern in the present study.

In addition to Harman’s single-factor test, common method bias was further assessed using the full collinearity variance inflation factor (VIF) approach, which is widely recommended in PLS-SEM studies. According to the recommended criterion, full collinearity VIF values below 3.3 indicate that common method bias is unlikely to threaten the validity of the findings. The results showed that all constructs exhibited VIF values below the threshold level, suggesting that common method bias was not a substantial concern in the present study (Table 2).

**Table 2:** Full collinearity VIF assessment.

Construct	Full collinearity VIF
SE	2.14
PW	2.32
EA	1.98
SD	2.45
CF	2.27
SC	2.51
EI	2.36

Therefore, the final dataset was considered suitable for further empirical analysis and hypothesis testing.

### Reliability analysis

The measurement model was evaluated in terms of indicator reliability, internal consistency reliability, convergent validity, and discriminant validity. These properties were assessed using factor loadings, Cronbach’s alpha, composite reliability (CR), average variance extracted (AVE), the Fornell Larcker criterion, the heterotrait monotrait ratio (HTMT), and Pearson correlation analysis (74).

As reported in Table 3, the factor loadings range from 0.642 to 0.889, indicating an acceptable level of indicator reliability. Although a limited number of items exhibit comparatively lower loadings, all retained indicators satisfy the minimum acceptable level for exploratory and model-development research (75). The values of Cronbach’s alpha range from 0.87 to 0.90, while the CR values range from 0.84 to 0.87, demonstrating satisfactory internal consistency reliability across all constructs. Similarly, the AVE values range from 0.65 to 0.72, exceeding the recommended threshold of 0.50 (76). These results confirm that the constructs possess adequate convergent validity.

**Table 3:** Reliability and validity.

Construct	Item	Mean	SD	Factor loading	VIF	Cronbach’s α	CR	AVE
Sports Education (SE)	SE1	4.18	0.92	0.821***	1.42	0.88	0.86	0.68
	SE2	4.05	0.95	0.762***	1.61			
	SE3	4.22	0.89	0.804***	1.48			
	SE4	4.30	0.87	0.691**	1.72			
	SE5	3.85	0.93	0.648	1.58			
Psychological Well-being (PW)	PW1	4.42	0.98	0.845***	1.50	0.87	0.85	0.66
	PW2	4.50	0.90	0.801***	1.63			
	PW3	4.38	0.94	0.712**	1.71			
	PW4	4.20	0.99	0.776***	1.60			
	PW5	4.30	0.91	0.695*	1.66			
Cognitive Focus (CF)	CF1	4.35	0.96	0.798***	1.58	0.88	0.86	0.67
	CF2	4.40	0.92	0.820***	1.49			
	CF3	4.60	0.88	0.851***	1.36			
	CF4	4.50	0.91	0.702**	1.65			
	CF5	4.32	0.97	0.667	1.73			
Social Development (SD)	SD1	4.50	0.89	0.812***	1.47	0.87	0.84	0.65
	SD2	4.42	0.91	0.789***	1.52			
	SD3	4.55	0.86	0.820***	1.45			
	SD4	4.60	0.88	0.695*	1.68			
	SD5	4.40	0.93	0.642	1.71			
Strategic Capacity (SC)	SC1	4.48	0.90	0.889***	1.28	0.89	0.87	0.72
	SC2	4.60	0.85	0.862***	1.34			
	SC3	4.55	0.88	0.738***	1.56			
	SC4	4.65	0.84	0.871***	1.30			
	SC5	4.30	0.92	0.701**	1.62			
Environmental Awareness (EA)	EA1	4.48	0.95	0.735***	1.61	0.90	0.86	0.69
	EA2	4.25	0.98	0.790***	1.52			
	EA3	4.70	0.90	0.842***	1.40			
	EA4	4.60	0.92	0.718**	1.60			
	EA5	4.28	0.96	0.676*	1.66			
Emotional Intelligence (EI)	EI1	4.38	0.94	0.812***	1.48	0.88	0.85	0.66
	EI2	4.45	0.91	0.798***	1.52			
	EI3	4.50	0.89	0.835***	1.42			
	EI4	4.42	0.93	0.690**	1.63			
	EI5	4.30	0.95	0.652	1.70			

***p < 0.01, *p < 0.1, and **p < 0.05.

Convergent validity reflects the degree to which indicators of a specific construct share a high proportion of common variance (77). In the present study, the minimum AVE value is 0.65 for Social Development, whereas the maximum AVE value is 0.72 for Strategic Capacity, thereby satisfying the accepted criterion for convergent validity (78). In addition, multicollinearity was assessed through variance inflation factor (VIF) values. The VIF values range from 1.28 to 1.73, which are substantially below the critical threshold of 3.0 (79). This finding indicates the absence of multicollinearity among the indicators and confirms that the observed variables are statistically distinct.

Discriminant validity was first examined using the Fornell–Larcker criterion (80). As shown in Table 4, the diagonal elements represent the square root of AVE for each construct. The results indicate that, for all constructs, the square root of AVE exceeds the corresponding inter-construct correlations. For instance, the square root of AVE for Sports Education is 0.85, which is higher than its correlations with Psychological Well-being (0.55), Environmental Awareness (0.50), Social Development (0.60), Cognitive Focus (0.57), Strategic Capacity (0.58), and Emotional Intelligence (0.54). Similar patterns are observed for the remaining constructs. These findings provide initial evidence of satisfactory discriminant validity (81).

**Table 4:** A Fornell–Larcker method for discriminant validity.

Construct	SE	PW	EA	SD	CF	SC	EI
SE	0.85						
PW	0.55***	0.86					
EA	0.50**	0.48*	0.87				
SD	0.60***	0.52***	0.45	0.85			
CF	0.57***	0.59***	0.51**	0.55***	0.86		
SC	0.58***	0.61***	0.50**	0.62***	0.63***	0.88	
EI	0.54**	0.65***	0.47	0.57***	0.60***	0.61***	0.84

***p < 0.01, *p < 0.1, and **p < 0.05.

To further substantiate discriminant validity, the heterotrait–monotrait ratio (HTMT) was also examined (82). The HTMT values reported in Table 5 range from 0.60 to 0.76, all of which remain below the recommended threshold of 0.90 (83). This result indicates that the constructs are empirically distinguishable from one another and that discriminant validity is adequately established.

**Table 5:** HTMT Approach.

Construct	SE	PW	EA	SD	CF	SC	EI
SE	–	0.68	0.63	0.71	0.69	0.70	0.67
PW	0.68	–	0.61	0.66	0.72	0.74	0.76
EA	0.63	0.61	–	0.60	0.62	0.64	0.63
SD	0.71	0.66	0.60	–	0.67	0.69	0.68
CF	0.69	0.72	0.62	0.67	–	0.73	0.71
SC	0.70	0.74	0.64	0.69	0.73	–	0.72
EI	0.67	0.76	0.63	0.68	0.71	0.72	–

The combined evidence from Tables 4, 5 demonstrates that the measurement model satisfies the requirements of discriminant validity. Specifically, the square roots of AVE exceed the inter-construct correlations, and all HTMT values remain within the acceptable range. Accordingly, the latent constructs included in the model may be regarded as conceptually and statistically distinct.

Following the assessment of reliability and validity, Pearson correlation analysis was performed to examine the magnitude and direction of the relationships among the study constructs (84). As presented in Table 6, all correlation coefficients are positive, indicating positive associations among the variables. Sports Education is moderately correlated with Social Development (r = 0.60), Strategic Capacity (r = 0.59), Cognitive Focus (r = 0.58), and Psychological Well-being (r = 0.57), suggesting that higher engagement in sports education is associated with improved psychological, cognitive, social, and strategic outcomes. Psychological Well-being exhibits its strongest correlations with Emotional Intelligence (r = 0.65), Strategic Capacity (r = 0.63), and Cognitive Focus (r = 0.62), indicating that mental and emotional functioning is closely associated with higher-order developmental outcomes. Environmental Awareness is positively associated with Sports Education (r = 0.53) and Psychological Well-being (r = 0.51), reflecting meaningful but moderate relationships. Social Development shows a relatively strong association with Strategic Capacity (r = 0.61), while Emotional Intelligence is positively correlated with Psychological Well-being (r = 0.65), Strategic Capacity (r = 0.62), and Cognitive Focus (r = 0.60), underscoring its relevance to psychological, cognitive, and social dimensions.

**Table 6:** Pearson correlation.

Construct	SE	PW	EA	SD	CF	SC	EI
SE	1.00						
PW	0.57	1.00					
EA	0.53	0.51	1.00				
SD	0.60	0.55	0.48	1.00			
CF	0.58	0.62	0.50	0.56	1.00		
SC	0.59	0.63	0.49	0.61	0.64	1.00	
EI	0.55	0.65	0.47	0.57	0.60	0.62	1.00

Overall, the findings reported in Tables 3–6 indicate that the measurement model demonstrates acceptable reliability, convergent validity, discriminant validity, and correlational adequacy. Furthermore, the absence of excessively high correlations suggests that multicollinearity is not a substantial concern. Therefore, the measurement model is considered statistically adequate for subsequent structural model assessment and hypothesis testing.

## Results

Data analysis was conducted using SmartPLS 4.0 through Partial Least Squares Structural Equation Modeling (PLS-SEM) (85). After establishing the reliability, convergent validity, discriminant validity, and correlation structure of the constructs, the next step involved evaluating the structural model. PLS-SEM was considered appropriate because it enables the simultaneous assessment of complex structural relationships among multiple latent constructs and is well suited for predictive and model-development research. In addition, the structural model was assessed using the coefficient of determination (R^2^), predictive relevance (Q^2^), effect size (f^2^), and the significance of the path coefficients obtained through bootstrapping (86).

Higher path coefficients indicate stronger relationships between the exogenous and endogenous constructs, while the coefficient of determination reflects the extent to which the endogenous variables are explained by the model (87). Predictive relevance was evaluated using Q^2^ values, and the significance of the direct and indirect effects was examined through *t*-values (88). Effect sizes were also considered in order to assess the substantive contribution of each predictor to the model (89). The results for the structural model are presented in Table 7, whereas the structural parameter estimates are reported in Table 6.

**Table 7:** Structural model.

Independent variable	R^2^	R^2^ adjusted	Interpretation
PW	0.54	0.52	Substantial
EA	0.58	0.57	Substantial
SD	0.46	0.45	Moderate
CF	0.55	0.54	Substantial
SC	0.63	0.61	Substantial

Dependent Variable	Q^2^ Value	R^2^ adjusted	Interpretation
PW	0.34		Moderate to high predictive relevance
EA	0.31		Moderate
SD	0.39		High predictive relevance
CF	0.37		High predictive relevance
SC	0.42		High predictive relevance

The structural model results presented in Table 7 indicate that the model has satisfactory explanatory power across the endogenous constructs. The R^2^ values show that Psychological Well-being (PW) has an explanatory power of 0.54, Environmental Awareness (EA) has 0.58, Social Development (SD) has 0.46, Cognitive Focus (CF) has 0.55, and Strategic Capacity (SC) has 0.63. The adjusted R^2^ values are also close to the corresponding R^2^ values, which suggests consistency in model estimation. Based on these values, the model demonstrates substantial explanatory power for PW, EA, CF, and SC, while the explanatory power for SD may be regarded as moderate (90).

The predictive relevance of the structural model was further assessed using Q^2^ values. The results show that all endogenous constructs have Q^2^ values greater than zero, confirming that the model possesses predictive relevance (91). Specifically, PW has a Q^2^ value of 0.34, EA has 0.31, SD has 0.39, CF has 0.37, and SC has 0.42. These findings indicate that the model has moderate to high predictive relevance, with the strongest predictive relevance observed for Strategic Capacity and Social Development (92). Overall, the results of R^2^ and Q^2^ collectively indicate that the proposed model has satisfactory explanatory and predictive capability.

The comparatively stronger explanatory power observed for Strategic Capacity and Environmental Awareness suggests that sports education plays an important role in shaping higher-order behavioral and developmental outcomes. Sports participation often involves planning, teamwork, discipline, tactical adaptation, and exposure to structured environments, which may directly strengthen strategic thinking and responsible behavioral orientation. Similarly, environmental awareness may develop through collective participation and increased sensitivity toward shared physical and social environments.

The mediation findings further indicate that Psychological Well-being and Strategic Capacity function as partial mediators because Sports Education continues to exert significant direct effects on Cognitive Focus and Social Development. This suggests that sports education influences these outcomes through both direct experiential mechanisms and indirect psychological and strategic pathways. The coexistence of significant direct and indirect effects therefore reflects the multidimensional nature of sports education within the proposed framework.

The structural parameter estimates are presented in Table 7. The results indicate that the majority of the proposed hypotheses are supported. Sports Education exerts a significant and positive effect on Social Development (H1: *β* = 0.42, t = 5.32), Psychological Well-being (H2: *β* = 0.38, t = 4.95), Environmental Awareness (H3: *β* = 0.45, t = 7.40), Cognitive Focus (H4: *β* = 0.43, t = 5.05), and Strategic Capacity (H5: *β* = 0.40, t = 5.18). These findings suggest that sports education plays a substantial role in improving social, psychological, environmental, cognitive, and strategic outcomes.

The strongest direct relationship among these paths is observed for H3, indicating that Sports Education has a particularly meaningful contribution to Environmental Awareness. Similarly, the positive and significant effects of Sports Education on Social Development, Cognitive Focus, and Strategic Capacity further confirm the central role of sports education in promoting multidimensional individual development.

The results also reveal that Psychological Well-being has a significant positive impact on Cognitive Focus (H6: *β* = 0.35, t = 4.60), while Strategic Capacity has a significant positive effect on Social Development (H7: *β* = 0.33, t = 3.72). These findings indicate that psychological well-being enhances cognitive functioning, whereas strategic capacity contributes meaningfully to social development. The effect sizes for these relationships remain within the moderate range (93), further supporting their substantive significance in the model.

With regard to mediation, the findings indicate that Psychological Well-being mediates the relationship between Sports Education and Cognitive Focus (H8: *β* = 0.27, t = 3.35), while Strategic Capacity mediates the relationship between Sports Education and Social Development (H9: *β* = 0.23, t = 2.68). Both mediation hypotheses are statistically supported (94), suggesting that Sports Education influences Cognitive Focus and Social Development not only directly, but also indirectly through improvements in Psychological Well-being and Strategic Capacity, respectively.

The inclusion of Emotional Intelligence as an additional construct also provides important findings. Emotional Intelligence shows a significant positive effect on Psychological Well-being (H10: *β* = 0.36, t = 4.82) and Cognitive Focus (H11: *β* = 0.30, t = 3.55). These results indicate that emotional intelligence enhances both psychological and cognitive functioning. However, the effect of Emotional Intelligence on Social Development (H12: *β* = 0.21, t = 1.95) is not statistically supported, and therefore H12 is rejected. This result suggests that while emotional intelligence is closely related to psychological well-being and cognitive focus, its direct contribution to social development may be comparatively weaker within the context of the present model.

The effect size (f^2^) values presented in Table 8 were interpreted in accordance with Cohen’s (1988) recommended thresholds, where values of 0.02, 0.15, and 0.35 indicate small, medium, and large effect sizes, respectively. The results demonstrate that the majority of the structural relationships exhibit medium effect sizes. Specifically, Sports Education showed medium effects on Social Development (f^2^ = 0.24), Psychological Well-being (f^2^ = 0.26), Environmental Awareness (f^2^ = 0.30), Cognitive Focus (f^2^ = 0.25), and Strategic Capacity (f^2^ = 0.27). Similarly, Psychological Well-being had a medium effect on Cognitive Focus (f^2^ = 0.22), while Strategic Capacity demonstrated a medium effect on Social Development (f^2^ = 0.20). Emotional Intelligence also exhibited medium effects on Psychological Well-being (f^2^ = 0.23) and Cognitive Focus (f^2^ = 0.19). In contrast, the effect of Emotional Intelligence on Social Development (f^2^ = 0.14) remained below the medium threshold and corresponded with the non-significant path coefficient. Overall, the findings indicate that Sports Education and the associated mediating constructs exert practically meaningful effects on the endogenous variables within the proposed model.

**Table 8:** Structural parameter estimate.

Hypotheses	*β*	*t*-values	SD	f^2^	Result
H1	0.42***	5.32	0.079	0.24	Yes
H2	0.38***	4.95	0.076	0.26	Yes
H3	0.45***	7.40	0.061	0.30	Yes
H4	0.43***	5.05	0.083	0.25	Yes
H5	0.40***	5.18	0.081	0.27	Yes
H6	0.35***	4.60	0.080	0.22	Yes
H7	0.33**	3.72	0.084	0.20	Yes
H8	0.27**	3.35	0.087		Yes
H9	0.23*	2.68	0.092		Yes
H10	0.36***	4.82	0.078	0.23	Yes
H11	0.30**	3.55	0.085	0.19	Yes
H12	0.21	1.95	0.094	0.14	No

***p < 0.01, *p < 0.1, and **p < 0.05.

Overall, the structural model results provide substantial support for the proposed conceptual framework. The findings confirm that Sports Education is a key determinant of Psychological Well-being, Environmental Awareness, Social Development, Cognitive Focus, and Strategic Capacity. In addition, Psychological Well-being and Strategic Capacity serve as important mediating mechanisms, while Emotional Intelligence contributes significantly to psychological and cognitive outcomes. Taken together, these results indicate that the model has satisfactory explanatory strength and provides meaningful empirical support for the majority of the proposed hypotheses.

**Figure 2:**
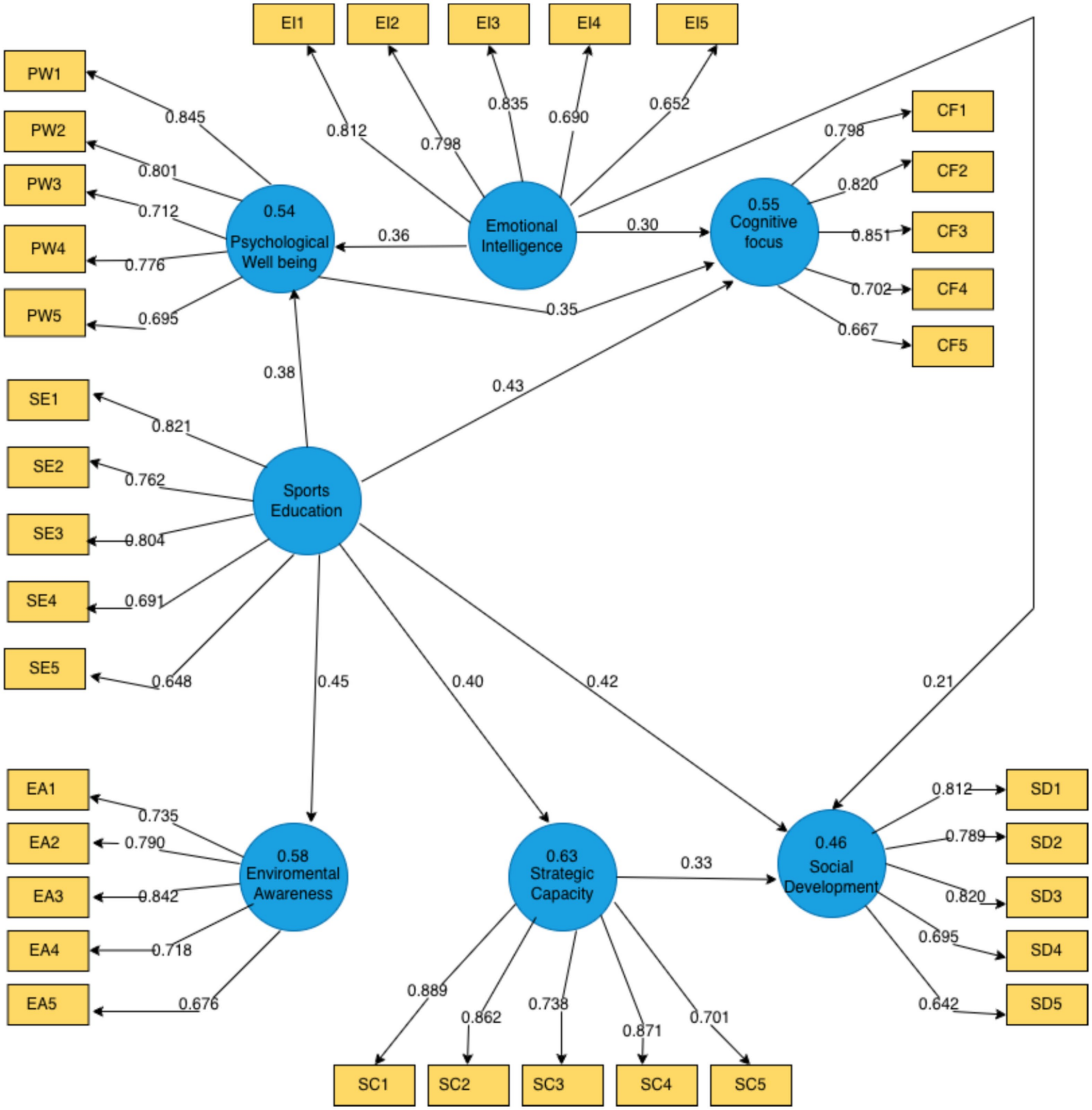
Structural model.

Figure 2 illustrates the structural model of the study, including the standardized path coefficients, outer loadings, and R^2^ values of the endogenous constructs. The figure shows that Sports Education has positive effects on Psychological Well-being, Environmental Awareness, Cognitive Focus, Strategic Capacity, and Social Development. It also indicates that Emotional Intelligence positively influences Psychological Well-being and Cognitive Focus, while its effect on Social Development is weaker. Overall, the figure confirms the proposed structural relationships among the study variables.

## Discussion

The findings of the present study provide substantial empirical support for the proposition that sports education is a significant determinant of multiple developmental outcomes, including social development, psychological well-being, environmental awareness, cognitive focus, and strategic capacity. The structural model results demonstrate that sports education exerts positive and statistically significant effects on these core constructs, thereby reinforcing its multidimensional value in educational and social contexts. In addition, the results highlight the mediating roles of psychological well-being and strategic capacity, while also demonstrating the relevance of emotional intelligence as an additional explanatory construct for psychological and cognitive outcomes. Taken together, these findings suggest that sports education should not be viewed merely as a physical or extracurricular activity, but rather as an important developmental mechanism with psychological, cognitive, social, strategic, and environmental implications.

Although Sports Education demonstrated a significant positive effect on Environmental Awareness, the comparatively moderate effect size suggests that environmental sensitivity may depend on the nature and context of sports participation. Individuals engaged in outdoor sports activities may experience stronger environmental awareness because of their direct interaction with natural surroundings, public spaces, and ecological conditions. In contrast, participants involved primarily in indoor or highly structured sports environments may have fewer opportunities for direct environmental engagement. The findings therefore suggest that environmental awareness may not emerge automatically through sports participation alone, but may also depend on the integration of sustainability-oriented practices and environmental learning within sports programs.

The support for H1 indicates that sports education has a positive effect on social development. This finding suggests that participation in sports education enhances individuals’ social interaction, teamwork, cooperation, and communication. Sports settings often require coordination with others, collective problem-solving, and adherence to shared rules and responsibilities, all of which contribute to the development of social competencies (95). The result implies that sports education can strengthen interpersonal relationships and promote social integration by bringing individuals into structured and cooperative environments. It also suggests that sports education may contribute to community cohesion by encouraging participation, mutual respect, and collaborative engagement (96). In this respect, the findings confirm that sports education extends beyond physical development and meaningfully contributes to broader social outcomes.

The impact of sports activities on social development is not limited to communication within teams. Studies conducted particularly on international students demonstrate that sports make significant contributions to socialization, cultural adaptation, and social integration processes (97).

The support for H2 further demonstrates that sports education positively influences psychological well-being. This indicates that sports education can reduce stress, improve self-esteem, and foster emotional resilience. The structured nature of sports participation may provide individuals with a sense of achievement, discipline, belonging, and emotional balance, all of which are closely associated with better mental well-being (4). The positive association between sports education and psychological well-being suggests that regular engagement in sports-related educational activities may provide an effective mechanism for coping with stress and maintaining emotional stability (4). These results reinforce the importance of sports education as a psychologically beneficial domain that contributes to holistic development rather than merely physical fitness.

The positive and significant effect of sports education on environmental awareness, as reflected in H3, indicates that sports education may also contribute to individuals’ sensitivity toward environmental issues. This result implies that involvement in sports, particularly where outdoor participation, discipline, and organized awareness are present, may foster appreciation for natural environments and sustainable practices (32). Sports education may indirectly cultivate environmental consciousness by exposing participants to settings in which cleanliness, responsibility, and stewardship are encouraged (98). The strength of this relationship in the present study suggests that environmental awareness can emerge as an important outcome of sports education, thereby expanding the traditional understanding of sports as a purely physical or social activity. The result also highlights the possibility that educational institutions and sports programs may serve as platforms for embedding environmentally responsible values (99).

The findings also confirm H4, which states that sports education positively influences cognitive focus. This indicates that sports education contributes to concentration, attentional control, and information processing ability (100). Participation in sports often requires individuals to maintain alertness, anticipate actions, process information rapidly, and respond accurately under pressure (101). Such demands may sharpen cognitive efficiency and improve mental discipline (102). The positive relationship between sports education and cognitive focus therefore suggests that physical engagement and cognitive development are closely interconnected. This result implies that sports education can contribute not only to physical competence but also to academic and intellectual functioning by strengthening attention and mental clarity (103).

Similarly, the support for H5 indicates that sports education has a significant positive effect on strategic capacity. This finding suggests that sports education contributes to the development of planning, decision-making, adaptability, and problem-solving ability (104). In structured sports contexts, individuals are often required to evaluate dynamic situations, formulate tactical responses, and adjust their actions in accordance with changing conditions (105). These repeated experiences may cultivate strategic thinking and increase the capacity to act effectively in both competitive and non-competitive settings (106). The substantial effect of sports education on strategic capacity indicates that sports-based learning may serve as an important foundation for developing higher-order competencies that are transferable to educational, professional, and social environments.

The results also support H6, showing that psychological well-being has a positive effect on cognitive focus. This indicates that emotionally stable individuals with lower levels of stress and better mental balance are more likely to maintain attention and perform effectively in cognitively demanding situations (107). The finding is consistent with the view that psychological well-being provides an internal condition that facilitates concentration, mental clarity, and sustained engagement (108). In other words, individuals who are psychologically healthier may be better able to allocate cognitive resources efficiently, avoid distraction, and remain attentive to tasks. This result further demonstrates that psychological functioning is closely related to cognitive performance and supports the broader idea that emotional and cognitive processes are interdependent (109).

The support for H7 shows that strategic capacity positively affects social development. This suggests that individuals with stronger planning ability, better decision-making, and greater adaptability are also more capable of functioning effectively in social environments (110). Strategic capacity may enhance social development by improving one’s ability to organize activities, collaborate with others, respond constructively to challenges, and contribute to collective goals (111). This result indicates that social development is not solely a direct outcome of interpersonal interaction, but is also facilitated by cognitive and strategic skills that enable more effective participation in group and community settings. Consequently, strategic capacity appears to be an important bridge between individual competence and social functioning (112).

The mediation findings further enrich the interpretation of the model. The support for H8 indicates that psychological well-being mediates the relationship between sports education and cognitive focus. This suggests that sports education improves cognitive focus not only directly, but also indirectly by enhancing psychological well-being (96). In practical terms, this means that one important pathway through which sports education contributes to attention and concentration is the improvement of emotional stability, stress management, and mental balance (4). The significance of this mediation effect indicates that psychological well-being is an important explanatory mechanism in the relationship between sports participation and cognitive development (10). At the same time, because the direct path from sports education to cognitive focus also remains significant, the mediation may be interpreted as partial rather than full, suggesting that sports education influences cognitive focus through multiple pathways (113).

The support for H9 similarly indicates that strategic capacity mediates the relationship between sports education and social development. This implies that sports education contributes to social development not only through direct social interaction, but also through the development of strategic competence (96). In this pathway, sports education enhances planning, adaptability, and decision-making, and these strategic qualities in turn strengthen individuals’ ability to function effectively in social contexts (33). This finding is important because it highlights a deeper developmental mechanism underlying social outcomes. Rather than treating social development as a purely interpersonal phenomenon, the model suggests that social competence is also shaped by cognitive and strategic abilities cultivated through sports education (114).

The inclusion of emotional intelligence adds another important dimension to the model. The support for H10 indicates that emotional intelligence has a positive effect on psychological well-being. This suggests that individuals who are better able to understand, regulate, and manage emotions are more likely to maintain emotional stability and psychological balance (48). Emotional intelligence may reduce vulnerability to stress and enhance self-awareness, thereby supporting overall well-being (115). This finding reinforces the view that emotional capabilities are central to psychological functioning and that emotional intelligence constitutes a meaningful predictor of mental health-related outcomes (116).

The support for H11 demonstrates that emotional intelligence also has a significant positive effect on cognitive focus. This result suggests that individuals who can regulate emotions more effectively are better positioned to avoid emotional distraction and maintain concentration (107). Emotional intelligence may therefore support cognitive performance by helping individuals remain composed, attentive, and mentally organized in challenging situations (117). The finding indicates that emotional intelligence contributes not only to psychological stability but also to cognitive efficiency, thereby strengthening the broader developmental framework proposed in the study (118).

In contrast, H12 was not supported, indicating that emotional intelligence does not have a statistically significant direct effect on social development in the present model. This result suggests that the relationship between emotional intelligence and social development may be weaker or more complex than expected (119). It is possible that emotional intelligence contributes to social development indirectly through other variables, or that social development in the present context is more strongly shaped by direct experiences in sports education and strategic capacity than by emotional intelligence alone (120). The lack of support for H12 does not diminish the importance of emotional intelligence, but rather indicates that its influence may be more specific to psychological and cognitive domains than to direct social outcomes within this model.

From a broader perspective, the findings of the present study underscore the value of adopting an integrative framework in examining the effects of sports education. The results show that sports education is associated with a wide range of outcomes spanning psychological, cognitive, social, strategic, and environmental domains. This multidimensional influence suggests that sports education should be treated as a meaningful component of educational and developmental policy (4). Institutions that incorporate sports education into their curriculum and co-curricular activities may be able to promote not only physical well-being, but also mental health, attentional capacity, strategic thinking, environmental consciousness, and social development (121).

The findings also carry important practical implications. For educational institutions, the results imply that investment in structured sports education programs may generate broader developmental benefits than are often recognized (122). For policymakers, the findings suggest that sports education can be used as a strategic tool to enhance youth development, social cohesion, and well-being (96). For practitioners and program designers, the model highlights the importance of designing sports education initiatives that also cultivate emotional regulation, strategic thinking, and reflective engagement, thereby maximizing their developmental impact. The positive roles of psychological well-being and strategic capacity as mediators further suggest that interventions should not only promote participation in sports, but also foster emotionally supportive and strategically engaging sports environments (123).

Overall, the discussion of the structural model indicates that sports education is a powerful explanatory factor in the development of multiple important outcomes. The findings support the central role of sports education in enhancing psychological well-being, environmental awareness, cognitive focus, strategic capacity, and social development, while also highlighting the significance of emotional intelligence for psychological and cognitive functioning. At the same time, the unsupported relationship between emotional intelligence and social development points to the need for further inquiry into the contextual and indirect mechanisms through which emotional capacities may influence social outcomes. These results provide a strong foundation for future theoretical refinement and empirical investigation.

Although the study included demographic variables such as gender, educational level, and residential background (urban/rural), multi-group analysis (MGA) was not conducted in the present research. Previous studies have suggested that sports participation and its developmental outcomes may differ across demographic groups. In particular, Kayantaş et al. (7) reported significant gender-based differences in physical activity outcomes, with stronger effect sizes observed among males. Therefore, it is possible that the structural relationships examined in the present model may vary across gender, educational level, or residential context. Future studies are encouraged to apply multi-group analysis techniques within PLS-SEM in order to compare path differences across demographic groups and to provide a more nuanced understanding of how sports education influences psychological, cognitive, social, and strategic outcomes across diverse populations.

### Implications of the study

#### Theoretical implications

The present study contributes to the growing body of literature examining the multidimensional role of sports education by integrating psychological, social, cognitive, strategic, environmental, and emotional perspectives into a single framework. The findings demonstrate that sports education is not limited to physical development; rather, it exerts meaningful effects on psychological well-being, social development, cognitive focus, strategic capacity, and environmental awareness. In this way, the study broadens the conceptual understanding of sports education as a multidimensional developmental mechanism.

The positive relationship between sports education and social development provides support for broader social and relational perspectives, particularly those emphasizing cooperation, communication, and collective engagement. The findings indicate that sports education contributes to teamwork, social interaction, and interpersonal effectiveness, thereby reinforcing the view that structured sports participation can strengthen social cohesion and community-oriented behavior. This extends the literature by showing that sports education can serve as an important socialization platform within educational and developmental settings.

The significant effect of sports education on psychological well-being also supports theoretical arguments that organized physical activity contributes to emotional stability, self-esteem, and resilience. The findings suggest that sports education satisfies important psychological needs by promoting competence, discipline, belonging, and emotional balance. As such, the study strengthens the theoretical linkage between sports participation and positive mental functioning, while also demonstrating that psychological well-being acts as an important explanatory mechanism in the relationship between sports education and cognitive outcomes.

The results further contribute to the literature on cognitive development by showing that sports education and psychological well-being both enhance cognitive focus. This finding supports the proposition that physical engagement and emotional stability jointly influence attention, concentration, and information processing. The study therefore advances theoretical understanding by linking sports education with executive and attentional functioning in a more integrated manner. Similarly, the significant effect of sports education on strategic capacity extends existing work on planning, decision-making, and adaptability by showing that sports-related experiences contribute to the development of higher-order strategic skills.

An additional theoretical contribution of the study lies in the incorporation of emotional intelligence as an extra construct in the model. The findings reveal that emotional intelligence has significant positive effects on psychological well-being and cognitive focus, suggesting that emotional regulation and self-awareness are important psychological resources in the sports education context. Although emotional intelligence did not show a statistically significant direct effect on social development, its significant relationships with psychological and cognitive outcomes indicate that it remains an important variable for extending existing sports education models. This provides a useful basis for future research seeking to integrate emotional and behavioral dimensions more systematically into sports education frameworks.

The study also contributes to mediation-based explanations of sports education outcomes. The significant mediating roles of psychological well-being and strategic capacity suggest that the influence of sports education on cognitive focus and social development operates not only directly, but also indirectly through internal psychological and strategic mechanisms. These findings enrich the theoretical understanding of how sports education generates developmental outcomes and demonstrate that both affective and strategic processes should be taken into account when examining the broader impact of sports participation.

#### Practical implications

Beyond its theoretical contributions, the study offers several practical implications for educators, policymakers, institutions, and sports organizations.

Educational institutions should recognize sports education as an important component of holistic development rather than treating it solely as a physical or extracurricular activity. Since sports education significantly improves psychological well-being, cognitive focus, social development, and strategic capacity, schools, colleges, and universities should integrate structured sports programs more systematically into their curricula. Such programs should emphasize not only physical activity, but also teamwork, discipline, strategic thinking, and reflective learning.

The positive effect of sports education on psychological well-being suggests that sports-based interventions may be used as a supportive tool for promoting mental health in educational settings. Institutions should therefore encourage regular participation in organized sports activities as part of student well-being initiatives. Programs designed to enhance emotional resilience, reduce stress, and improve self-confidence through physical engagement may be especially beneficial in high-pressure academic environments.

The findings also indicate that sports education contributes to cognitive focus and strategic capacity. This implies that sports-based activities can support not only physical and emotional growth, but also attentional control, planning ability, and decision-making skills. Educational institutions and training centers may therefore use sports education as a complementary mechanism for improving concentration, mental discipline, and adaptive problem-solving. This is particularly relevant for environments in which students and young adults are expected to perform under pressure and respond effectively to changing demands.

The strong relationship between sports education and environmental awareness further suggests that sports programs can be used to promote responsible environmental attitudes. Sports institutions, schools, and community organizations should incorporate sustainability-oriented practices into sports education, such as clean-campus campaigns, environmentally responsible event management, recycling activities, and awareness-building around green spaces and public resources. In this way, sports education can serve as a channel for reinforcing ecological responsibility alongside personal development.

The findings regarding social development indicate that sports education can also be used to strengthen community engagement and inclusion. Policymakers and community organizations should support accessible sports programs for diverse populations, including young people, underserved communities, and socially marginalized groups. Structured sports activities can help foster communication, cooperation, social trust, and collective participation, thereby contributing to stronger communities and more inclusive social environments.

The additional role of emotional intelligence in the model also has practical implications. Since emotional intelligence significantly improves psychological well-being and cognitive focus, educators and trainers should consider integrating emotional skills development into sports education programs. Activities that encourage emotional regulation, empathy, self-awareness, and constructive communication may further enhance the developmental impact of sports participation. Although emotional intelligence did not show a direct effect on social development in the present study, its indirect relevance suggests that it may still strengthen the broader quality of participation and performance in sports-based settings.

Overall, the practical implications of the study suggest that sports education should be approached as a multidimensional developmental tool capable of enhancing psychological, cognitive, social, strategic, and environmental outcomes. Institutions and policymakers that invest in structured and inclusive sports education programs are likely to generate benefits that extend well beyond physical health alone.

### Limitations and future research

While the present study offers useful insights into the multidimensional role of sports education, several limitations should be acknowledged.

- The study used a cross-sectional design, which limits the ability to establish causal relationships among the variables because the temporal direction of the relationships cannot be fully confirmed.
- The findings are based on self-reported data, which may involve response bias, social desirability bias, and common method variance, thereby potentially affecting the precision of the measured relationships. Future studies may use objective indicators, such as cognitive performance tests, behavioral measures, or external assessments, to improve validity.
- Another limitation relates to the consistently positive and statistically significant relationships observed across the proposed hypotheses. Although these findings are theoretically aligned with prior literature on sports education and developmental outcomes, the strength of the relationships may also reflect certain methodological factors, including sample homogeneity, self-reported perceptions, and potential social desirability tendencies among respondents. Since the participants shared relatively similar educational and sports-related backgrounds, variability in responses may have been reduced, potentially contributing to stronger positive associations among the constructs. Therefore, the findings should be interpreted with appropriate caution. Future studies are encouraged to utilize more heterogeneous samples, longitudinal designs, and multi-source data collection approaches to further validate the robustness and generalizability of the observed relationships.
- The sample was drawn from a specific context using convenience sampling, which may restrict the broader generalizability of the findings across different cultural, institutional, and socioeconomic environments. In addition, the study relied on a convenience sampling approach, which may limit the representativeness of the sample and reduce external validity. Although efforts were made to recruit respondents from diverse demographic backgrounds through stratified outreach, important subgroup differences such as age categories, sports branches, professional versus amateur participation, and cultural backgrounds were not examined separately. These factors may influence psychological, cognitive, and social outcomes associated with sports education. Therefore, future studies are encouraged to apply probability-based sampling techniques or conduct multi-group comparative analyses in order to improve generalizability and provide deeper contextual understanding across different participant groups.

Future research should include broader and more diverse demographic groups to test the model across different settings and populations.

- Although the model includes psychological well-being, environmental awareness, social development, cognitive focus, strategic capacity, and emotional intelligence, other relevant variables may also influence these relationships. Future studies may extend the framework by incorporating constructs such as motivation, resilience, academic performance, or institutional support.
- Emotional intelligence was included as an additional construct, but its direct effect on social development was not statistically significant. Future research may further explore whether emotional intelligence affects social development indirectly through other factors such as empathy, communication, or team cohesion.
- The mediating roles of psychological well-being and strategic capacity provide an initial explanatory framework. Future studies may benefit from using mixed-method approaches, comparative designs, or more detailed theoretical perspectives to better understand the contextual and causal processes involved.
- Future research may benefit from longitudinal designs, probability-based sampling techniques, and cross-cultural comparative approaches to improve causal interpretation and external validity.

## Conclusion

The present study offers an original contribution to the literature by developing and empirically validating a multidimensional public health framework linking sports education with psychological well-being, cognitive focus, social development, strategic capacity, environmental awareness, and emotional intelligence. Unlike prior studies that have largely examined these outcomes separately, the current research integrates these dimensions into a unified structural model and demonstrates the direct and indirect mechanisms through which sports education contributes to holistic human development.

This study examined the relationships among sports education, psychological well-being, social development, cognitive focus, strategic capacity, environmental awareness, and emotional intelligence within the proposed research framework. The analysis was conducted through structural equation modeling, and the findings emphasize the significant role of sports education in shaping multiple dimensions of individual development. The results demonstrate that sports education has a strong positive influence on social development, psychological well-being, environmental awareness, cognitive focus, and strategic capacity. These findings indicate that sports education should be understood not merely as a physical activity domain, but as a broader developmental mechanism that contributes to psychological, cognitive, social, and environmental outcomes.

An important conclusion of the study is that sports education positively contributes to psychological well-being and social development. Participation in sports-related educational activities appears to improve emotional stability, self-esteem, and interpersonal functioning, while also strengthening teamwork, communication, and social interaction. These results support the view that sports education promotes both personal growth and broader social cohesion. The findings also confirm that sports education significantly enhances cognitive focus, suggesting that involvement in organized sports helps individuals maintain attention, improve concentration, and strengthen mental clarity. This indicates that sports education may serve as an effective means of improving cognitive discipline and attentional control, which are valuable for academic, professional, and everyday performance.

The study further finds that strategic capacity plays an important role in the model, both as a direct outcome of sports education and as a meaningful predictor of social development. This highlights the importance of sports education in developing planning ability, adaptability, and decision-making skills. The mediating role of strategic capacity in the relationship between sports education and social development suggests that sports education contributes to social outcomes not only through direct interaction, but also through the development of higher-order strategic skills. In a similar way, psychological well-being was found to mediate the relationship between sports education and cognitive focus, indicating that the psychological benefits of sports participation contribute significantly to improved attentional and cognitive outcomes. These findings show that sports education influences development through both direct and indirect pathways.

Another important finding concerns the role of environmental awareness. The results indicate that sports education has a positive effect on environmental awareness, suggesting that participation in sports may encourage greater appreciation of clean, safe, and sustainable surroundings. This expands the scope of sports education by showing that its influence may extend beyond personal and social development to include environmental sensitivity and responsible attitudes. In addition, emotional intelligence was incorporated as an extra construct in the model and was found to have significant positive effects on psychological well-being and cognitive focus. This suggests that emotional regulation and self-awareness strengthen the psychological and cognitive benefits associated with sports education. However, the direct relationship between emotional intelligence and social development was not supported, indicating that this relationship may be weaker or operate through indirect pathways.

The findings underline the importance of integrating sports education into broader educational and developmental frameworks. By enhancing psychological well-being, cognitive focus, strategic capacity, environmental awareness, and social development, sports education offers benefits that extend well beyond physical health. These results suggest that educators, institutions, and policymakers should give greater attention to sports education as a means of promoting well-rounded development. Investment in structured sports education programs may therefore contribute to the formation of healthier, more capable, and more socially engaged individuals who are better prepared to contribute positively to society.

## Data Availability

The original contributions presented in the study are included in the article/Supplementary material, further inquiries can be directed to the corresponding author.
