# Supplementary material for: The sports-education-health nexus: assessing psychological, cognitive, and social outcomes in a public health framework
Source: Front Public Health. 2026 Jun 15;14:1859935. doi: 10.3389/fpubh.2026.1859935 (PMC13310990; doi:10.3389/fpubh.2026.1859935)
Supplement: Supplementary file 1 [file Supplementary_file_1.docx]

**Appendix**

## Sports Education (SE)

Adapted from Yang et al. (2024)

- SE1: Sports education activities improve my physical and personal development.
- SE2: Participation in sports education enhances my teamwork abilities.
- SE3: Sports education helps me develop discipline and responsibility.
- SE4: The sports education programs I participate in are well organized and effective.
- SE5: Sports education contributes positively to my overall well-being.

## Psychological Well-being (PW)

Adapted from Park et al. (2022)

- PW1: Participation in sports activities helps me reduce stress.
- PW2: I feel emotionally stable when participating in sports-related activities.
- PW3: Sports activities improve my self-confidence.
- PW4: I generally feel psychologically healthy and balanced.
- PW5: Sports participation helps me maintain a positive mindset.

## Social Development (SD)

Adapted from Liqiang et al. (2024)

- SD1: Sports participation improves my communication skills.
- SD2: Sports activities help me cooperate effectively with others.
- SD3: Participation in sports strengthens my social relationships.
- SD4: Sports education encourages teamwork and collaboration.
- SD5: Sports participation increases my sense of belonging within groups.

## Cognitive Focus (CF)

Adapted from Walters et al. (2025)

- CF1: Sports participation improves my concentration.
- CF2: I can maintain attention better during tasks after participating in sports activities.
- CF3: Sports activities help me stay mentally focused.
- CF4: Participation in sports improves my ability to process information efficiently.
- CF5: Sports education enhances my mental clarity during academic or daily activities.

## Strategic Capacity (SC)

Adapted from Müller et al. (2025)

- SC1: Sports participation improves my decision-making abilities.
- SC2: I am better able to plan and organize activities because of sports participation.
- SC3: Sports education enhances my problem-solving skills.
- SC4: Participation in sports helps me adapt to changing situations.
- SC5: Sports activities strengthen my strategic thinking abilities.

## Environmental Awareness (EA)

Adapted from Baltodano-Nontol et al. (2024)

- EA1: Sports activities increase my appreciation for natural environments.
- EA2: I am more aware of environmental sustainability because of sports participation.
- EA3: Sports education encourages environmentally responsible behavior.
- EA4: Participation in outdoor sports increases my concern for environmental protection.
- EA5: Sports-related activities motivate me to support eco-friendly practices.

## Emotional Intelligence (EI)

Adapted from Yang and Duan (2023)

- EI1: I can effectively manage my emotions in difficult situations.
- EI2: I am aware of my emotional reactions during sports activities.
- EI3: I can understan the emotions of others.
- EI4: I remain calm and emotionally balanced under pressure.
- EI5: I can regulate my emotions when interacting with others.
